# Secondary Versus Recurrent Central Nervous System Lymphoma: Evaluating the Similarities and Differences in a Single-Institution Cohort

**DOI:** 10.7759/cureus.87626

**Published:** 2025-07-09

**Authors:** Danielle A Bazer, Melanie Schweitzer, Ewa Zabrocka, Agnieszka Kowalska

**Affiliations:** 1 Neurology/Neuro-Oncology, Johns Hopkins University, Baltimore, USA; 2 Neurology, Stony Brook University, Stony Brook, USA; 3 Radiation Oncology, Stony Brook University Hospital, Stony Brook, USA

**Keywords:** case report series, diffuse large b-cell lymphoma (dlbcl), ebv negative, primary central nervous system lymphoma (pcnsl), recurrent central nervous system lymphoma, secondary central nervous system lymphoma

## Abstract

Background: Primary central nervous system lymphoma (PCNSL) is a rare malignancy that affects only a small number of individuals. A subset of these cases may later relapse as recurrent central nervous system lymphoma (RPCNSL), while systemic lymphomas may progress to secondary central nervous system lymphoma (SCNSL). Although these entities differ in origin, they often present with overlapping clinical and radiologic features, complicating diagnosis and management.

Objective: This study aims to compare the clinical presentations, treatment histories, lesion characteristics, and outcomes of patients with RPCNSL and SCNSL in a single-institution cohort in an attempt to identify key distinguishing features and shared patterns. Improved understanding of these similarities and differences may enhance diagnostic accuracy, guide treatment strategies, and inform surveillance approaches.

Methods: We conducted a retrospective chart review of six patients (three with RPCNSL, three with SCNSL) treated at our institution between 2010 and 2020. Demographic data, initial cancer diagnosis and treatment, central nervous system (CNS) involvement, imaging findings, clinical symptoms, and outcomes were analyzed.

Results: All RPCNSL lesions were solitary and localized to the frontal or temporal lobes, while SCNSL lesions were multifocal in two of three patients. Altered mental status was a common presenting symptom across both groups. Treatment regimens and timing of CNS involvement varied widely. All RPCNSL patients eventually transitioned to hospice care.

Conclusion: Despite differing pathogeneses, SCNSL and RPCNSL share overlapping clinical features that may obscure timely diagnosis. Recognizing their distinct patterns in lesion distribution, timing of CNS involvement, and treatment history is crucial for tailoring care and improving outcomes. Larger studies are needed to validate these findings and support the development of evidence-based guidelines.

## Introduction

Although rare, primary central nervous system lymphoma (PCNSL) affects 0.5 per 100,000 individuals [1,2]. It is estimated that 5% cases will develop into secondary central nervous system lymphoma (SCNSL) and 2-5% will evolve into recurrent central nervous system lymphoma (RPCNSL) with the diffuse large B-cell lymphoma (DLBCL) variant of RPCNSL [3,4]. With the adrenal involvement, relapse rates as high as 40% have been reported [3,4]. The incidence of both SCNSL and RPCNSL is increasing, and the prognosis of these patients remains poor despite advances in systemic therapy; it is anticipated that patients with SCNSL will die within a few months of the diagnosis [5,6].

The use of rituximab-based therapy has decreased the risk of central nervous system (CNS) recurrence, and common modalities for CNS prophylaxis include intrathecal methotrexate (with or without cytarabine and/or hydrocortisone) or intravenous high-dose methotrexate [4]. However, once a patient has developed SCNSL, there is a paucity of consensus guidelines due to a lack of randomized clinical trial data, prompting clinicians to rely on retrospective data and phase II studies [7].

The goal of this study was to compare clinical presentation, imaging characteristics, treatment regimens, and outcomes to identify distinguishing patterns between RPCNSL and SCNSL. We attempted to characterize early differentiators that may guide diagnosis, risk stratification, and treatment selection.

This abstract was presented at the Society for Neuro-Oncology Annual Meeting in November 2022.

## Materials and methods

Study design

This is a retrospective case series that relied on chart review to identify appropriate participants; the chart review commenced after institutional review board approval. All study participants were treated at our University Hospital between 2010 and 2020. The participant cases were divided based on RPCNSL, SCNSL, and PCNSL. We narrowed our inclusion criteria to include patients living with RPCNSL and SCNSL.

Inclusion and exclusion criteria

This study included patients with SCNSL and RPCNSL, DLBCL subtype. Our study required patients to be greater than 18 years of age. Patients were evaluated at our hospital from the years 2010-2020. It was mandatory that the patients have their initial MRI, work-up, and treatment plan established at our institution.

This study excluded patients with primary CNS lymphoma. Patients who developed CNS lymphoma as a complication of human immunodeficiency virus (HIV) were excluded from this study. Patients who were also Epstein-Barr virus (EBV) positive were excluded from this study as well. We excluded individuals who had their initial treatment and/or work-up at an outside institution.

Data collection

Patients were identified using the key terms “CNS lymphoma,” “secondary CNS lymphoma,” and “recurrent primary CNS lymphoma” as well as the ICD-10 codes associated with such diagnoses. The electronic medical record was also queried to include patients greater than 18 years of age from the years 2010-2020. Investigators then reviewed the generated list of patients and went through the individual patient charts to determine if the patients were appropriate for study inclusion.

Statistical analysis

Descriptive statistics were used to interpret the data. Variables included age of primary malignancy diagnosis, age of secondary malignancy diagnosis, initial treatment for PCNSL, presenting symptoms, location of the tumor, and outcomes. The investigators performed the descriptive statistical analyses.

Ethics statement

This study was approved by the university’s institutional review board in 2021 and received the following IRB number: IRB2021-00200. Written consent was waived by the institutional review board. The study complies with the Declaration of Helsinki.

## Results

Our series contained six patients, 3/6 with SCNSL and 3/6 with RPCNSL. All RPCNSL patients were male. In the SCNSL cohort, 2/3 were male. All patients in the study were Caucasian, HIV negative, and EBV negative. The average age at diagnosis of the first malignancy in patients with RPCNSL was 63 years (range, 56-71; median, 61 years), and the age at recurrence was 67 years (range, 63-71; median, 66 years). In SCNSL, the average age at initial diagnosis was 73 years (range, 65-77; median, 76 years), and the average age at secondary presentation was 76 years (range, 67-81; median, 80 years).

Initial PCNSL treatments for RPCSNL were steroids, R-CHOP (rituximab, cyclophosphamide, doxorubicin, vincristine, prednisone) with radiation, and DeAngelis Protocol (methotrexate, leucovorin, vincristine, procarbazine, whole brain radiation, dexamethasone, and cytarabine).

Initial malignancies for patients with SCNSL were DLBCL with renal involvement in two patients and atypical chronic lymphocytic leukemia (CLL) variant with 17p deletion in the remaining patient. The initial treatments for the patients who had DLBCL with renal involvement were stereotactic radiation as well as DA-EPOCH-R (dose-reduced etoposide, prednisone, vincristine, cyclophosphamide, doxorubicin, and rituximab) and R-CVP (rituximab, cyclophosphamide, vincristine, and prednisone). The patient with atypical CLL variant with 17p deletion was initially treated with ibrutinib, bendamustine, rituximab, and idelalisib, followed by R-CVP. 

**Figure 1:**
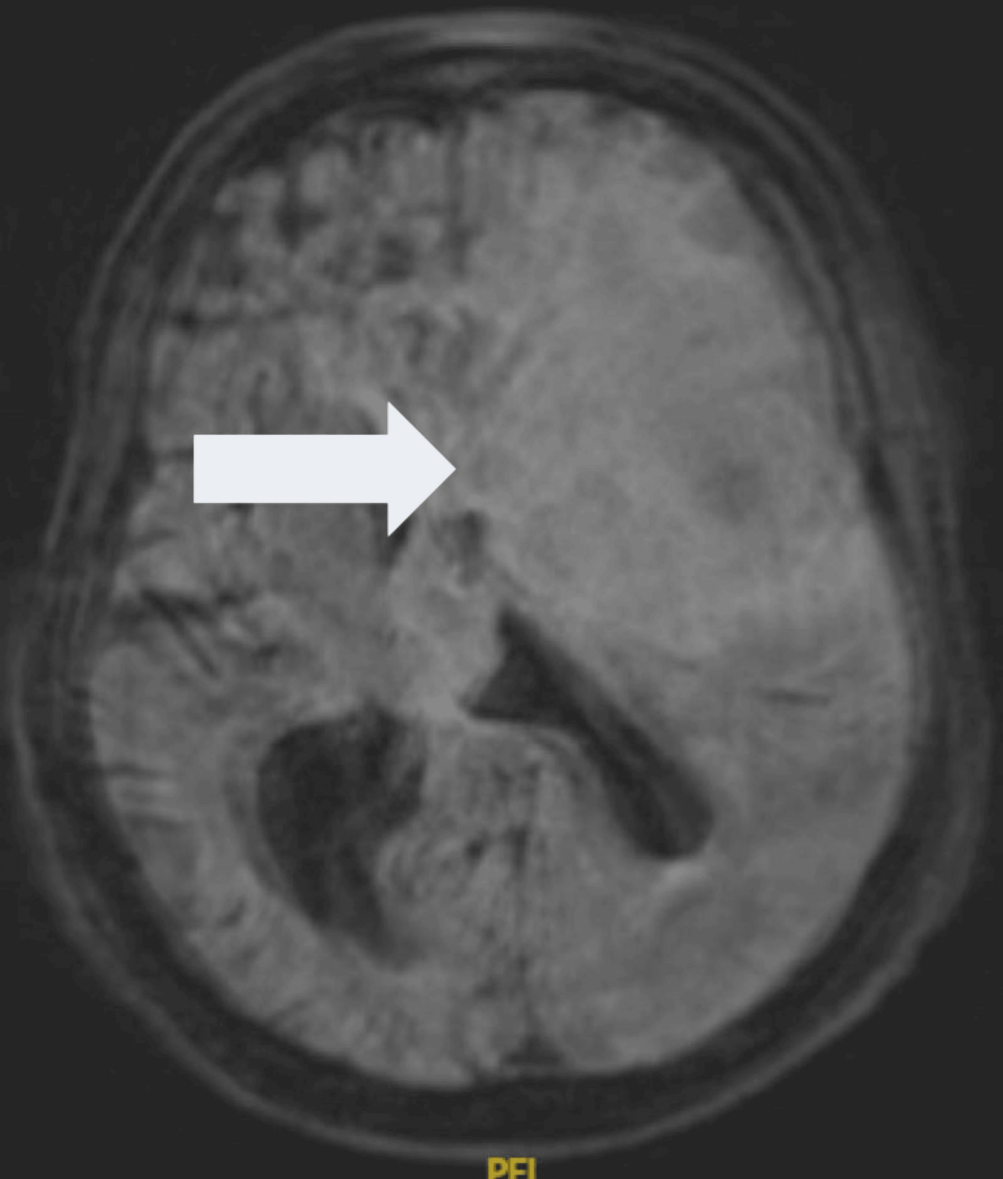
MRI of the brain of patient 1 with RPCNSL There is a large, heterogeneous, centrally necrotic mass in the left frontal lobe and basal ganglia region, with extensive surrounding vasogenic edema and mass effect causing effacement of the sulci. The mass extends into the lateral ventricles and involves the body and genu of the corpus callosum. RPCNSL = recurrent central nervous system lymphoma

**Figure 2:**
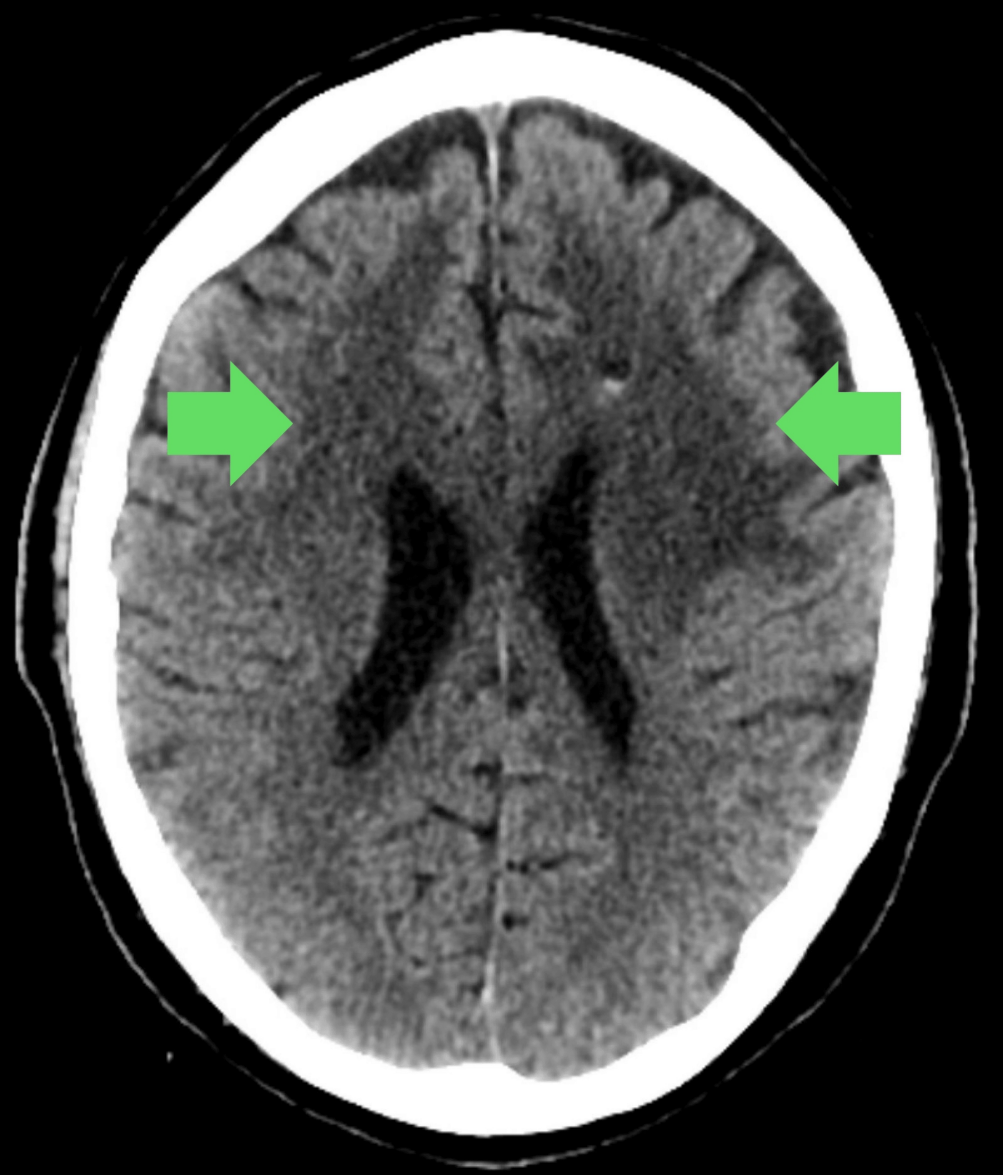
Head CT of patient 2 with RPCNSL Low-attenuation foci are present in the bilateral frontal lobes and centrum semiovale, crossing the midline. RPCNSL = recurrent central nervous system lymphoma

**Figure 3:**
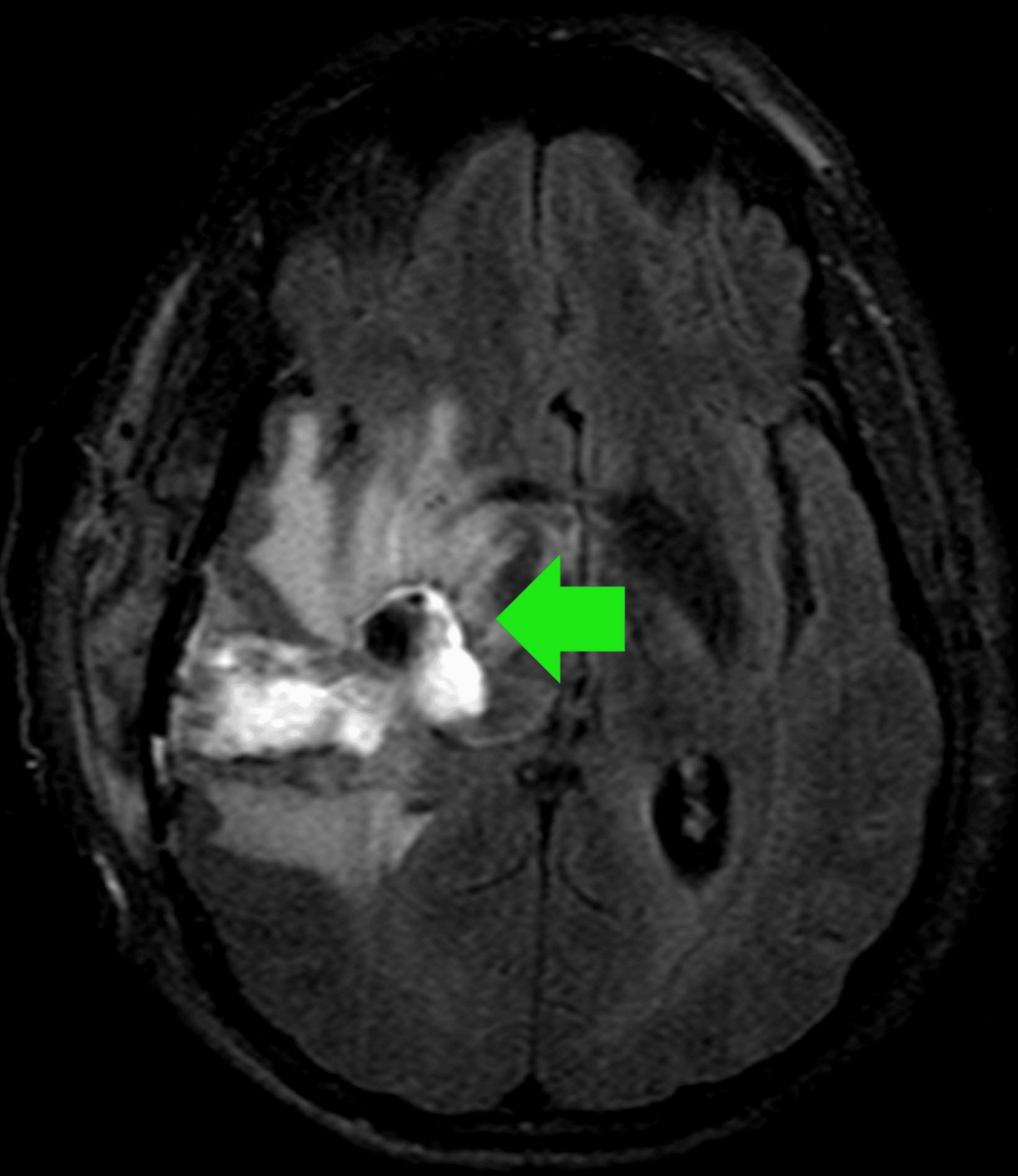
T2/FLAIR MRI of the brain in patient 3 with RPCNSL A heterogeneous mass is present in the medial temporal lobe, with surrounding vasogenic edema. RPCNSL = recurrent central nervous system lymphoma

**Figure 4:**
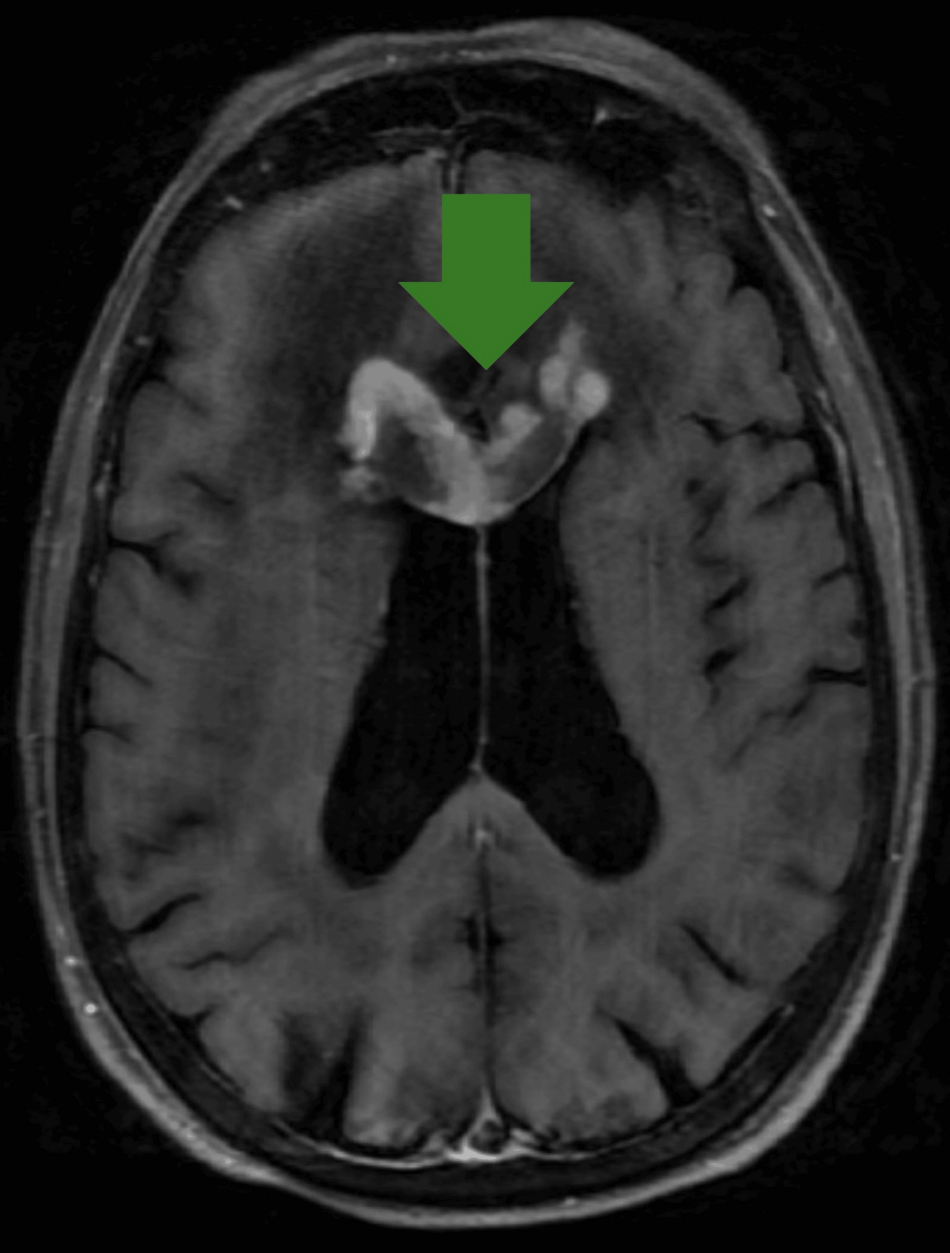
T1-weighted post-contrast MRI of the brain in patient 1 with SCNSL There is an enhancing lesion of the right genu of the corpus callosum. SCNSL = secondary central nervous system lymphoma

**Figure 5:**
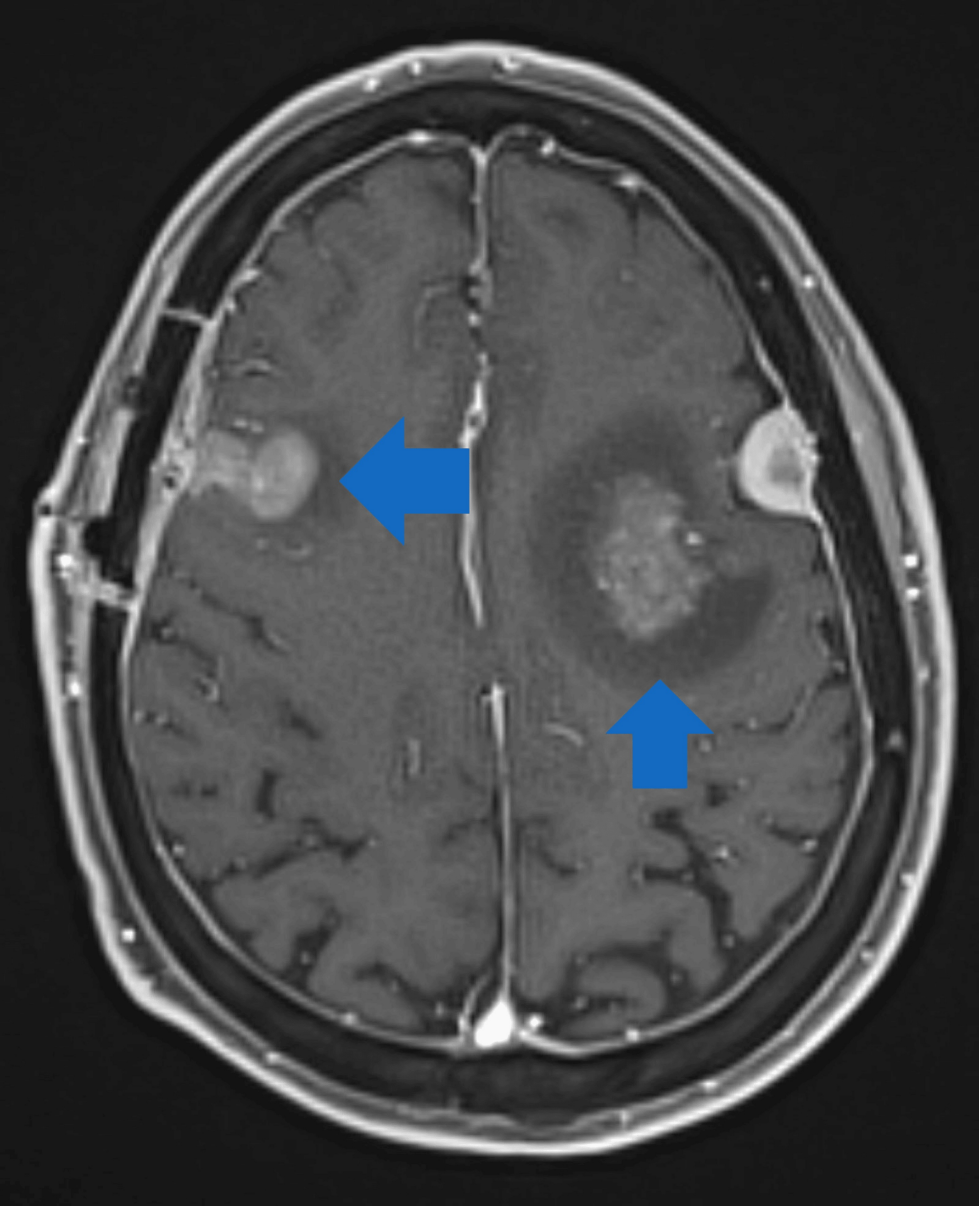
T1-weighted post-contrast MRI of the brain in patient 2 with SCNSL It shows a left posterior frontal lobe lesion involving the precentral gyrus, as well as a right frontal lobe lesion with a surrounding hemosiderin ring and associated vasogenic edema. SCNSL = secondary central nervous system lymphoma

**Figure 6:**
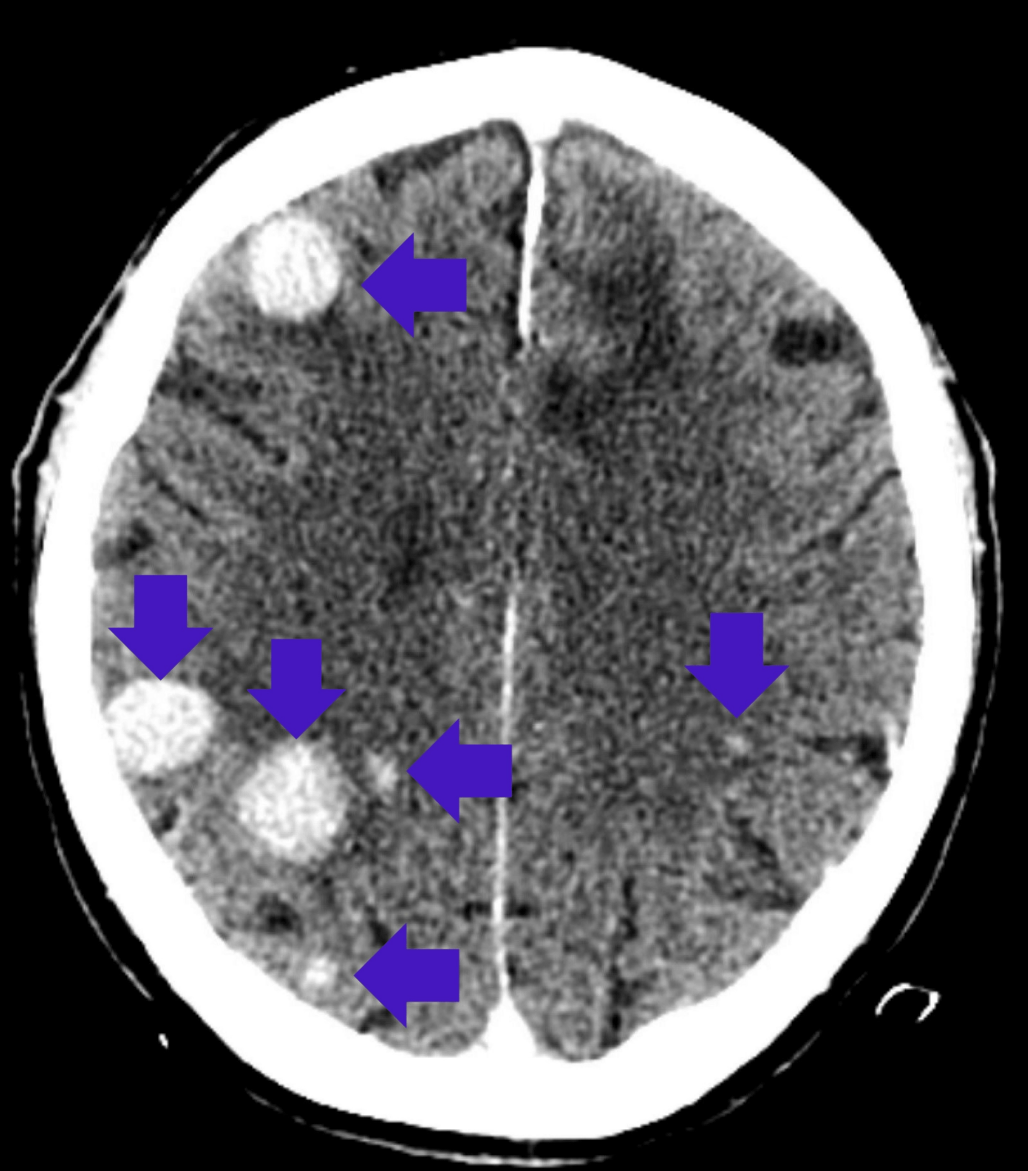
Contrast-enhanced head CT of patient 3 with SCNSL There are numerous hyperdense mass lesions in the bilateral cerebral hemispheres with contrast enhancement. SCNSL = secondary central nervous system lymphoma

All patients with RPCNSL presented with altered mental status and 2/3 SCNSL patients were altered upon presentation; one SCNSL patient was asymptomatic. The lesions within the RPCNSL cohort were all singular lesions, localized to the frontal and temporal lobes (Figures 1-3). Within the SCNSL cohort, 2/3 patients had multiple lesions in the frontal, parietal, temporal, and occipital lobes, whereas 1 patient had a single lesion in the genu of the corpus callosum (Figure 4-6). 

At recurrence, patients with PCNSL were treated with R-MVP (rituximab, methotrexate, procarbazine, vincristine); rituximab, methotrexate, and cytarabine; and rituximab, temozolomide, and cytarabine. The patients with SCNSL were treated with steroids and rituximab, DeAngelis Protocol, and high-dose methotrexate. The history and treatment course of all six patients are summarized in Table 1. 

**Table 1:** Individual patient characteristics and treatment courses SCNSL = secondary central nervous system lymphoma; RPCNSL = recurrent primary central nervous system lymphoma; M = male; F = female; AMS = altered mental status; PCNSL = primary central nervous system lymphoma; DLBCL = diffuse large B-cell lymphoma; R-MVP = rituximab, methotrexate, procarbazine, vincristine; R-CHOP = rituximab, cyclophosphamide, doxorubicin, vincristine, prednisone; HD-MTX = high-dose methotrexate; DeAngelis protocol = methotrexate, vincristine, procarbazine, leucovorin, dexamethasone, whole brain radiation, cytarabine; TMZ = temozolomide; DA-EPOCH-R = etoposide, prednisone, vincristine, cyclophosphamide, doxorubicin, rituximab; R-CVP = rituximab, cyclophosphamide, vincristine, prednisone; CLL = chronic lymphocytic leukemia; PFS = progression-free survival; OS = overall survival

Patient #	Sex	SCNSL or RPCNSL?	Age at Initial Presentation	Age at Recurrence/ Secondary Presentation	Presenting Symptom for RCNSL/SCNSL	Imaging at Presentation of SCNSL/RCNSL	Prior Cancer History	Treatment Received at Initial Presentation	Treatment Received at Recurrence/ Secondary Presentation	Outcome (Months)
1	M	RPCNSL	71	71	AMS, gait ataxia	MRI without contrast: Large heterogeneous, centrally necrotic mass with restricted diffusion, centered in the left frontal lobe and basal ganglia. Extensive surrounding vasogenic edema and mass effect with sulcal effacement. Extension into the lateral ventricles, involving the body and genu of the corpus callosum. Rightward midline shift (15 mm) with subfalcine herniation.	PCNSL	Steroids	R-MVP	PFS=14; OS=15
2	M	RPCNSL	56	66	AMS, frequent falls, left arm weakness, urinary incontinence	CT without contrast: Infiltrative low-attenuation foci in the bilateral frontal lobes and centrum semiovale, extending across the corpus callosum.	PCNSL	R-CHOP and radiation	Rituximab, HD-MTX, cytarabine	PFS=129; OS=137
3	M	RPCNSL	61	63	AMS, personality changes, right-sided weakness	CT with contrast: Marked solid enhancement in the right medial temporal region with surrounding vasogenic edema. Two small, ill-defined central and left hypodense foci. Compression of the right lateral ventricle and brainstem with leftward midline shift.	PCNSL	DeAngelis Protocol	Rituximab, TMZ, cytarabine	PFS=23; OS=28
4	F	SCNSL	77	81	AMS, starring spells	MRI with and without contrast: Extensive FLAIR hyperintensity in bilateral frontal lobes with heterogeneous enhancement and expansion of the genu and proximal body of the corpus callosum. Mass effect on the lateral ventricle. Ring-enhancing lesion in the right frontal lobe abutting the right lateral ventricle. Multiple homogeneously enhancing lesions in the left frontal lobe (largest 1.1 cm), also involving the corpus callosum.	DLBCL	Stereotactic radiation	Steroids, rituximab	PFS=unknown; outcome: lost to follow-up
5	M	SCNSL	65	67	Asymptomatic	MRI with and without contrast: Left frontal lobe mass with surrounding edema. Similar mass in the right frontal lobe with surrounding hemosiderin ring, edema, and overlying burr hole (biopsy site). Additional mass in the right occipital lobe and a smaller lesion in the left frontal lobe. Left frontal extra-axial, dural-based lesion. Associated local mass effect.	DLBCL	DA-EPOCH-R, R-CVP	DeAngelis Protocol	PFS=12; outcome= lost to follow-up
6	M	SCNSL	76	80	AMS, gait ataxia	CT with and without contrast: Numerous hyperdense mass lesions in bilateral cerebral hemispheres with homogeneous enhancement, consistent with lymphoma metastases. Partial effacement of the left frontal horn.	Atypical CLL variant with 17p deletion	Ibrutinib, bendamustine, rituximab, idelalisib. R-CVP	HD-MTX	PFS=12; outcome= lost to follow-up

All of the RPCNSL patients were ultimately transitioned to palliative care and all of the SCNSL patients were lost to follow-up (Table 2).

**Table 2:** Demographic characteristics and outcomes of the patients with both RCNSL and SCNSL RPCNSL = recurrent central nervous system lymphoma; SCNSL = secondary central nervous system lymphoma; R-MVP = rituximab, methotrexate, procarbazine, vincristine; AMS = altered mental status; DLBCL = diffuse large B-cell lymphoma; CLL = chronic lymphocytic leukemia

Variable	RCNSL	SCNSL
Number of Patients	3	3
Gender	2/3 male, 1/3 female	3/3 male
Race	3/3 Caucasian	3/3 Caucasian
Median Age of Initial Presentation	63 years	67 years
Median Age of Secondary Presentation	73 years	76 years
Primary Cancer	PCNSL	2/3 DLBCL with renal involvement; 1/3 atypical CLL variant with 17 q deletion
Primary Cancer Treatment at Recurrence	R-MVP; rituximab-methotrexate-cytarabine; rituximab-temozolomide-cyclophosphamide	Rituximab; DeAngelis Protocol, high-dose methotrexate
MRI Findings at Recurrence	3/3 with single lesions localized to the frontal and temporal lobes	2/3 had multiple lesions in the frontal, parietal, temporal, and occipital lobes. 1 patient had a single lesion in the genu of the corpus callosum
Presentation at Recurrence	3/3 AMS	2/3 AMS, 1/3 asymptomatic
Outcome	3/3 deceased	3/3 lost to follow-up

## Discussion

Despite limited case numbers, comparative data on SCNSL and RPCNSL are sparse. This case series is an attempt at a side-by-side comparison of these two rare CNS lymphoma presentations from a single institution, highlighting potential distinguishing features in clinical course, imaging, and outcomes that may aid in diagnostic and therapeutic decision-making.

Although differing in etiology, there are shared features among SCNSL and RPCNSL. In our cohort, five out of six patients presented with altered mental status. The majority (~75%) of RPCNSL patients are symptomatic at the time of relapse, with the most common symptoms including gait disorder, cognitive impairment, sensorimotor disorder, and balance issues [8]. The presentation is similar in the case of SCNSL [9]. Most relapses are confined to the central nervous system; however, a small number of relapses are isolated systemic relapses, and clinical symptoms occur early and vary. Although treatment is similar for both RPCNSL and SCNSL, the prognosis for RPCNSL is very poor [10,11].

The RPCNSL lesions were all singular and localized to the frontal and temporal lobes for our cohort. The lesions in the SCNSL cohort were more variable with 2/3 patients having multiple lesions and 1/3 patients having a single lesion in the corpus callosum. This is consistent with the work of Malikova et al., showing the presence of multiple lesions at the SCNSL diagnosis in over half of the patients [12]. Although SCNSL has been shown to present with leptomeningeal disease in 2/3 of patients, we have not observed it in our series [13,14]. This points to the heterogeneity of presentations, which illuminates a potential diagnostic challenge.

Although the literature delineates that rituximab-based therapy can decrease the risk of CNS recurrence in PCNSL patients [4], only two out of three patients with RPCNSL received a rituximab-based therapy as part of their initial regimen. Of the patients that received rituximab-based therapy for PCNSL, only one patient received CNS prophylaxis with methotrexate, which may contribute, in part, to why there was recurrent disease in the CNS. Yet, at recurrence, all patients received rituximab-based therapy for RPCNSL. Two out of three of the patients also received methotrexate, yet only one patient received both methotrexate and cytarabine in combination.

Similar to the diverse presentation of SCNSL, there was greater variety in the treatments delivered at initial presentation as well as at the time of secondary presentation. Our cohort is consistent with the current literature identifying that there is a predilection for genitourinary involvement [3,4]. In our cohort, among the patients with DLBCL and renal involvement, one was treated with systemic therapies (DA-EPOCH-R and R-CVP), while the other received stereotactic radiotherapy. The patient living with atypical CLL variant with 17p deletion also received R-CVP after pretreatment with ibrutinib, bendamustine, rituximab, and idelalisib. Similar to the RPCNSL cohort, two patients received a rituximab-based therapy for their initial cancer diagnosis. None of the patients with SCNSL received methotrexate as part of their initial cancer regimen. At the time of secondary presentation, only 1 patient was treated with a rituximab-based therapy, whereas the remaining 2 patients were treated with the DeAngelis Protocol and high-dose methotrexate. 

In our cohort for both RPCNSL and SCNSL, the median progression-free survival (PFS) was 14 months. The median PFS for the RPCNSL cohort was 23 months and 12 months for the SCNSL. The median overall survival was challenging to calculate, as all patients with SCNSL were lost to follow-up. Of the patients with RPCNSL, the mean overall survival was 28 months. Unfortunately, our cohort is consistent with the current literature describing the high rate of mortality at recurrence/secondary progression [5,6]. These data highlight the immense need to develop better evidence-based treatments to improve the survival of patients living with both SCNSL and RPCNSL.

Although our case series is small, it contributes to our greater understanding of both RPCNSL and SCNSL. It offers a direct clinical comparison between RPCNSL and SCNSL, identifying trends such as lesion multiplicity, symptom presentation, and treatment heterogeneity. Notably, all patients with RPCNSL had solitary lesions and uniformly poor outcomes, while SCNSL cases were more variable in both lesion distribution and response to treatment. These observations may support earlier recognition and tailored management strategies in future clinical practice or research.

There are some limitations to our study. The small sample size and limited follow-up data, due to the retrospective nature of this study, limit our ability to confidently generalize the results. Nevertheless, considering the rarity of RPCNSL and SCNSL diagnoses, and taking into account the paucity of large, prospective clinical studies investigating the nature of these diseases [11,15], we believe that our study is a valuable addition to the fund of knowledge on these entities.

## Conclusions

Despite the small sample size, our study highlights several key distinctions between RPCNSL and SCNSL. First, our study reveals a male and geriatric predominance for both RPCNSL as well as SCNSL. For the RPCNSL cohort, lesions were singular in all cases, whereas for SCNSL, the tumors had a predilection to be multifocal. All of the patients with RCNSL were transitioned to hospice. Thus, a high level of suspicion for a diagnosis of RPCNSL should be raised if there is a geriatric male patient with a history of primary CNS lymphoma with subsequent brain imaging suggesting a solitary lesion.

Unfortunately, our sample size is quite small; however, the trends we have identified can be hypothesis-generating and can guide further prospective, multi-institutional research that is much needed to better characterize prognostic factors, guide treatment decisions, and ultimately improve survival for patients with SCNSL and RPCNSL.
